# Bias in random forest variable importance measures: Illustrations, sources and a solution

**DOI:** 10.1186/1471-2105-8-25

**Published:** 2007-01-25

**Authors:** Carolin Strobl, Anne-Laure Boulesteix, Achim Zeileis, Torsten Hothorn

**Affiliations:** 1 Institut für Statistik, Ludwig-Maximilians-Universität München, Ludwigstr. 33, 80539 München, Germany; 2 Institut für medizinische Statistik und Epidemiologie, Technische Universität München, Ismaningerstr. 22, 81675 München, Germany; 3 Department für Statistik und Mathematik, Wirtschaftsuniversität Wien, Augasse 2-6, 1090 Wien, Austria; 4 Institut für Medizininformatik, Biometrie und Epidemiologie, Friedrich-Alexander-Universtität Erlangen-Nürnberg, Waldstr. 6, D-91054 Erlangen, Germany

## Abstract

**Background:**

Variable importance measures for random forests have been receiving increased attention as a means of variable selection in many classification tasks in bioinformatics and related scientific fields, for instance to select a subset of genetic markers relevant for the prediction of a certain disease. We show that random forest variable importance measures are a sensible means for variable selection in many applications, but are not reliable in situations where potential predictor variables vary in their scale of measurement or their number of categories. This is particularly important in genomics and computational biology, where predictors often include variables of different types, for example when predictors include both sequence data and continuous variables such as folding energy, or when amino acid sequence data show different numbers of categories.

**Results:**

Simulation studies are presented illustrating that, when random forest variable importance measures are used with data of varying types, the results are misleading because suboptimal predictor variables may be artificially preferred in variable selection. The two mechanisms underlying this deficiency are biased variable selection in the individual classification trees used to build the random forest on one hand, and effects induced by bootstrap sampling with replacement on the other hand.

**Conclusion:**

We propose to employ an alternative implementation of random forests, that provides unbiased variable selection in the individual classification trees. When this method is applied using subsampling without replacement, the resulting variable importance measures can be used reliably for variable selection even in situations where the potential predictor variables vary in their scale of measurement or their number of categories. The usage of both random forest algorithms and their variable importance measures in the R system for statistical computing is illustrated and documented thoroughly in an application re-analyzing data from a study on RNA editing. Therefore the suggested method can be applied straightforwardly by scientists in bioinformatics research.

## Background

In bioinformatics and related scientific fields, such as statistical genomics and genetic epidemiology, an important task is the prediction of a categorical response variable (such as the disease status of a patient or the properties of a molecule) based on a large number of predictors. The aim of this research is on one hand to predict the value of the response variable from the values of the predictors, i.e. to create a diagnostic tool, and on the other hand to reliably identify relevant predictors from a large set of candidate variables. From a statistical point of view, one of the challenges in identifying these relevant predictor variables is the so-called "small *n *large *p*" problem: Usual data sets in genomics often contain hundreds or thousands of genes or markers that serve as predictor variables *X*_1_,..., *X*_*p*_, but only for a comparatively small number *n *of subjects or tissue types.

Traditional statistical models used in clinical case control studies for predicting the disease status from selected predictor variables, such as logistic regression, are not suitable for "small *n *large *p*" problems [1,2]. A more appropriate approach from machine learning, that has been proposed recently for prediction and variable selection in various fields related to bioinformatics and computational biology, is the nonlinear and nonparametric random forest method [3]. It also provides variable importance measures for variable selection purposes.

Random forests have been successfully applied to various problems in, e.g., genetic epidemiology and microbiology in general within the last five years. Within a very short period of time, random forests have become a major data analysis tool, that performs well in comparison with many standard methods [2,4]. What has greatly contributed to the popularity of random forests is the fact that they can be applied to a wide range of prediction problems, even if they are nonlinear and involve complex high-order interaction effects, and that random forests produce variable importance measures for each predictor variable.

Applications of random forests in bioinformatics include large-scale association studies for complex genetic diseases, as e.g. Lunetta et al. [5] and Bureau et al. [1], who detect SNP-SNP interactions in the case-control context by means of computing a random forest variable importance measure for each polymorphism. A comparison of the performance of random forests and other classification methods for the analysis of gene expression data is presented by Díaz-Uriate and Alvarez de Andrés [4], who propose a new gene selection method based on random forests for sample classification with microarray data. We refer to [6-8] for other applications of the random forest methodology to microarray data.

Prediction of phenotypes based on amino acid or DNA sequence is another important area of application of random forests, since possibly involving many interactions. For example, Segal et al. [9] use random forests to predict the replication capacity of viruses, such as HIV-1, based on amino acid sequence from reverse transcriptase and protease. Cummings and Segal [10] link the rifampin resistance in *Mycobacterium tuberculosis *to a few amino acid positions in rpoB, whereas Cummings and Myers [11] predict C-to-U edited sites in plant mitochondrial RNA based on sequence regions flanking edited sites and a few other (continuous) parameters.

The random forest approach was shown to outperform six other methods in the prediction of protein interactions based on various biological features such as gene expression, gene ontology (GO) features and sequence data [12]. Other applications of random forests can be found in fields as different as quantitative structure-activity relationship (QSAR) modeling [13,14], nuclear magnetic resonance spectroscopy [15], landscape epidemiology [16] and medicine in general [17].

The scope of this paper is to show that the variable importance measures of Breiman's original random forest method [3], based on CART classification trees [18], are a sensible means for variable selection in many of these applications, but are not reliable in situations where potential predictor variables vary in their scale of measurement or their number of categories, as, e.g., when both genetic and environmental variables, individually and in interactions, are considered as potential predictors, or predictor variables of the same type vary in the number of categories present in a certain sample, as is often the case in genomics, bioinformatics and related disciplines.

Simulation studies are presented illustrating that variable selection with the variable importance measure of the original random forest method bears the risk that suboptimal predictor variables are artificially preferred in such scenarios.

In an extra section, further details and explanations of the statistical sources underlying the deficiency of the variable importance measures of the original random forest method, namely biased variable selection in the individual classification trees used to build the random forest and effects induced by bootstrap sampling with replacement, are given.

We propose to employ an alternative random forest method, the variable importance measure of which can be employed to reliably select relevant predictor variables in any data set. The performance of this method is compared to that of the original random forest method in simulation studies, and is illustrated by an application to the prediction of C-to-U edited sites in plant mitochondrial RNA, re-analyzing the data of [11] that were previously analyzed with the original random forest method.

## Methods

Here we focus on the use of random forests for classification tasks, rather than regression tasks, for instance for predicting the disease status from a set of selected genetic and environmental risk factors, or for predicting whether a site of interest is edited by means of neighboring sites and other predictor variables as in our application example.

Random forests are an ensemble method that combines several individual classification trees in the following way: From the original sample several bootstrap samples are drawn, and an unpruned classification tree is fit to each bootstrap sample. The variable selection for each split in the classification tree is conducted only from a small random subset of predictor variables, so that the "small *n *large *p*" problem is avoided. From the complete forest the status of the response variable is predicted as an average or majority vote of the predictions of all trees.

Random forests can highly increase the prediction accuracy as compared to individual classification trees, because the ensemble adjusts for the instability of the individual trees induced by small changes in the learning sample, that impairs the prediction accuracy in test samples. However, the interpretability of a random forest is not as straightforward as that of an individual classification tree, where the influence of a predictor variable directly corresponds to its position in the tree. Thus, alternative measures for variable importance are required for the interpretation of random forests.

### Random forest variable importance measures

A naive variable importance measure to use in tree-based ensemble methods is to merely count the number of times each variable is selected by all individual trees in the ensemble.

More elaborate variable importance measures incorporate a (weighted) mean of the individual trees' improvement in the splitting criterion produced by each variable [19]. An example for such a measure in classification is the "Gini importance" available in random forest implementations. The "Gini importance" describes the improvement in the "Gini gain" splitting criterion.

The most advanced variable importance measure available in random forests is the "permutation accuracy importance" measure. Its rationale is the following: By randomly permuting the predictor variable *X*_*j*_, its original association with the response *Y *is broken. When the permuted variable *X*_*j*_, together with the remaining unpermuted predictor variables, is used to predict the response, the prediction accuracy (i.e. the number of observations classified correctly) decreases substantially, if the original variable *X*_*j *_was associated with the response. Thus, a reasonable measure for variable importance is the difference in prediction accuracy before and after permuting *X*_*j*_.

For variable selection purposes the advantage of the random forest permutation accuracy importance measure as compared to univariate screening methods is that it covers the impact of each predictor variable individually as well as in multivariate interactions with other predictor variables. For example, Lunetta et al. [5] find that genetic markers relevant in interactions with other markers or environmental variables can be detected more efficiently by means of random forests than by means of univariate screening methods like Fisher's exact test.

The Gini importance and the permutation accuracy importance measures are employed as variable selection criteria in many recent studies in various disciplines related to bioinformatics, as outlined in the background section. Therefore we want to investigate their reliability as variable importance measures in different scenarios.

In the simulation studies presented in the next section, we compare the behavior of all three random forest variable importance measures, namely the number of times each variable is selected by all individual trees in the ensemble (termed "selection frequency" in the following), the "Gini importance" and the permutation accuracy importance measure (termed "permutation importance" in the following).

### Simulation studies

The reference implementation of Breiman's original random forest method [3] is available in the R system for statistical computing [20] via the randomForest add-on package by Liaw and Wiener [21,22]. The behavior of the selection frequency, the Gini importance and the permutation importance of the randomForest function is explored in a simulation design where potential predictor variables vary in their scale of measurement and number of categories.

As an alternative, we propose to use the new random forest function cforest available in the R add-on package party [23] in such scenarios. In contrast to randomForest, the cforest function creates random forests not from CART classification trees based on the Gini split criterion [18], that are known to prefer variables with, e.g., more categories in variable selection [18,24-28], but from unbiased classification trees based on a conditional inference framework [29]. The problem of biased variable selection in classification trees is covered more thoroughly in a separate section below.

Since the cforest function does not employ the Gini criterion, we investigate the behavior of the Gini importance for the randomForest function only. The selection frequency and the permutation importance is studied for both functions randomForest and cforest in two ways: Either the individual trees are built on bootstrap samples of the original sample size *n *drawn with replacement, as suggested in [3], or on subsamples drawn without replacement.

For sampling without replacement the subsample size here is set to 0.632 times the original sample size *n*, because in bootstrap sampling with replacement about 63.2% of the data end up in the bootstrap sample. Other fractions for the subsample size are possible, for instance 0.5 as suggested by Friedman and Hall [30]. Subsampling as an alternative to bootstrap sampling in aggregating, e.g., individual classification trees is investigated further by Bühlmann and Yu [31], who also coin the term "subagging" as an abbreviation for "subsample aggregating" as opposed to "bagging" for "bootstrap aggregating". Politis, Romano and Wolf [32] show that, for statistical inference in general, subsampling works under weaker assumptions than bootstrap sampling and even in situations when bootstrap sampling fails.

The simulation design used throughout this paper represents a scenario where a binary response variable *Y *is supposed to be predicted from a set of potential predictor variables that vary in their scale of measurement and number of categories. The first predictor variable *X*_1 _is continuous, while the other predictor variables *X*_2_,..., *X*_5 _are categorical (on a nominal scale of measurement) with their number of categories between two and up to twenty. The simulation designs of both studies are summarized in Tables 1 and 2. The sample size for all simulation studies was set to *n *= 120.

**Table 1:** Simulation design for the simulation studies – predictor variables

Predictor variables	
*X*_1_	~*N*(0, 1)
*X*_2_	~*M*(2)
*X*_3_	~*M*(4)
*X*_4_	~*M*(10)
*X*_5_	~*M*(20)

The predictor variables are sampled independently from the following distributions. *N*(0, 1) stands for the standard normal distribution, *M*(*k*) stands for the multinomial distribution with values in {0,..., *k *- 1} and equal probabilities (discrete uniform distribution on {0,..., *k *- 1}), *B*(*p*) stands for the binomial distribution (Bernoulli distribution) with probability *p*, thus *M*(2) equals *B*(0.5).

**Table 2:** Simulation design for the simulation studies – response variable

	Response variable	
null case	*Y*	~*B*(0.5)
power case	*Y*|*X*_2 _= 1	~*B*(0.5 - *relevance*)
	*Y*|*X*_2 _= 2	~*B*(0.5 + *relevance*)

The response variable is sampled from binomial (Bernoulli) distributions. The degree of dependence between the response *Y *and *X*_2 _is regulated by the probability *p *of the binomial distribution *B*(*p*) of *Y *conditional on *X*_2_, with the *relevance *parameter taking values in {0.05, 0.1, 0.15, 0.2} to model different degrees of dependence.

In the first simulation study, the so-called null case, none of the predictor variables is informative for the response, i.e. all predictor variables and the response are sampled independently. In this situation a sensible variable importance measure should not prefer any one predictor variable over any other.

In the second simulation study, the so-called power case, the predictor variable *X*_2 _is informative for the response, i.e. the distribution of the response depends on the value of this predictor variable. The degree of dependence between the informative predictor variable *X*_2 _and the response *Y *is regulated by the *relevance *parameter of the conditional distribution of *Y *given *X*_2 _(cf. Table 2). We will later display results for different values of the *relevance *parameter indicating different degrees of dependence between *X*_2 _and *Y*. In the power case, a sensible variable importance measure should be able to distinguish the informative predictor variable from its uninformative competitors, and even more so with increasing degree of dependence.

## Results and discussion

Our simulation studies show that for the randomForest function all three variable importance measures are unreliable, and the Gini importance is most strongly biased. For the cforest function reliable results can be achieved both with the selection frequency and the permutation importance if the function is used together with subsampling without replacement. Otherwise the measures are biased as well.

### Results of the null case simulation study

**Figure 1:**
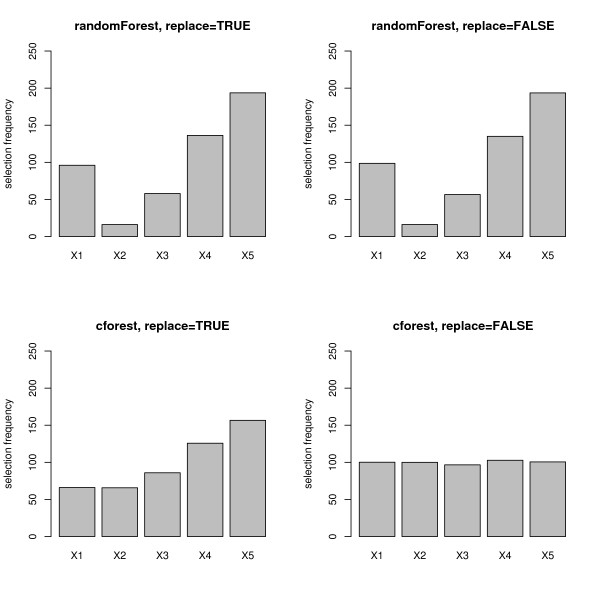
**Results of the null case study – variable selection frequency**. Mean variable selection frequencies for the null case, where none of the predictor variables is informative. The plots in the top row display the frequencies when the randomForest function is used, the bottom row when the cforest function is used. The left column corresponds to bootstrap sampling with replacement, the right column to subsampling without replacement.

In the null case, when all predictor variables are equally uninformative, the selection frequencies as well as the Gini importance and the permutation importance of all predictor variables are supposed to be equal. However, as presented in Figure 1, the mean selection frequencies (over 1000 simulation runs) of the predictor variables differ substantially when the randomForest function (cf. top row in Figure 1) or the cforest function with bootstrap sampling (cf. bottom row, left plot in Figure 1) are used. Variables with more categories are obviously preferred. Only when the cforest function is used together with subsampling without replacement (cf. bottom row, right plot in Figure 1) are the variable selection frequencies for the uninformative predictor variables equally low as desired.

It is obvious that variable importance cannot be represented reliably by the selection frequencies, that can be considered as very basic variable importance measures, if the potential predictor variables vary in their scale of measurement or number of categories when the randomForest function or the cforest function with bootstrap sampling is used.

**Figure 2:**
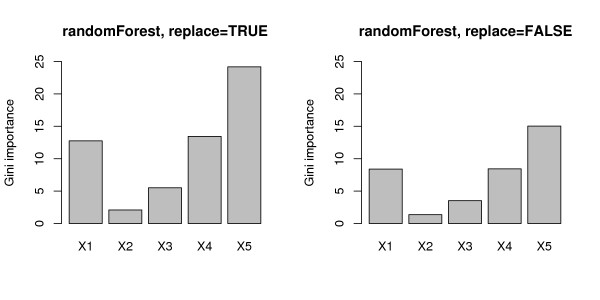
**Results of the null case study – Gini importance**. Mean Gini importance for the null case, where none of the predictor variables is informative. The left plot corresponds to bootstrap sampling with replacement, the right plot to subsampling without replacement.

The mean Gini importance (over 1000 simulation runs), that is displayed in Figure 2, is biased even stronger. Like the selection frequencies for the randomForest function (cf. top row in Figure 1) the Gini importance shows a strong preference for variables with many categories and the continuous variable, the statistical sources of which are explained in the section on variable selection bias in classification trees below. We conclude that the Gini importance cannot be used to reliably measure variable importance in this situation either.

We now consider the more advanced permutation importance measure. We find that here an effect of the scale of measurement or number of categories of the potential predictor variables is less obvious but still severely affects the reliability and interpretability of the variable importance measure.

**Figure 3:**
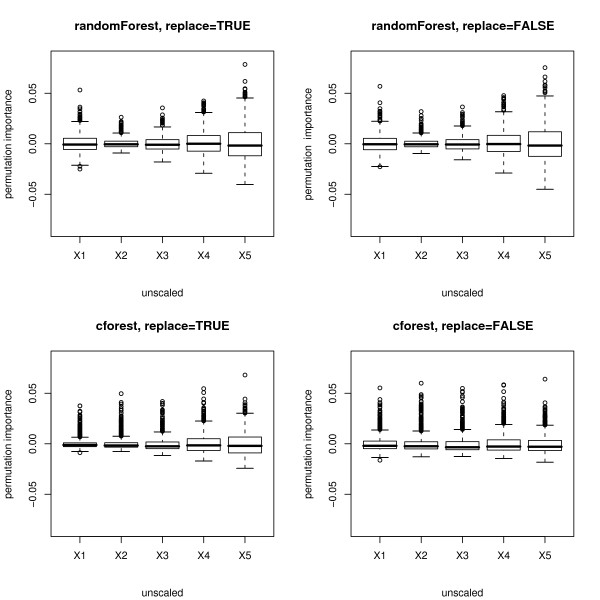
**Results of the null case study – unscaled permutation importance**. Distributions of the unscaled permutation importance measures for the null case, where none of the predictor variables is informative. The plots in the top row display the distributions when the randomForest function is used, the bottom row when the cforest function is used. The left column corresponds to bootstrap sampling with replacement, the right column to subsampling without replacement.

Figure 3 shows boxplots of the distributions (over 1000 simulation runs) of the permutation importance measures of both functions for the null case. The plots in the top row again display the distribution when the randomForest function is used, the bottom row when the cforest function is used. The left column of plots displays the distributions when bootstrap sampling is conducted with replacement, while the right column displays the distributions when subsampling is conducted without replacement.

**Figure 4:**
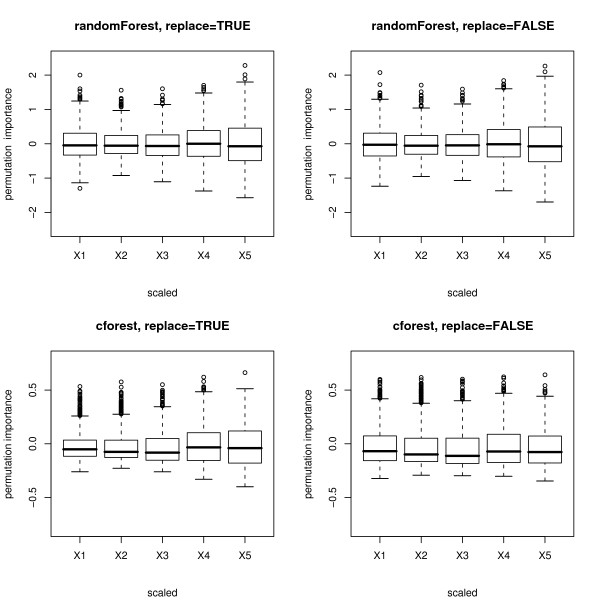
**Results of the null case study – scaled permutation importance**. Distributions of the scaled permutation importance measures for the null case, where none of the predictor variables is informative. The plots in the top row display the distributions when the randomForest function is used, the bottom row when the cforest function is used. The left column corresponds to bootstrap sampling with replacement, the right column to subsampling without replacement.

Figure 4 shows boxplots of the distributions of the scaled version of the permutation importance measures of both functions, incorporating the standard deviation of the measures.

The scaled variable importance is the default output of the randomForest function. However, it has been noted, e.g., by Díaz-Uriate and Alvarez de Andrés [4] in their supplementary material, that the scaled variable importance of the randomForest function depends on the number of trees grown in the random forest. (In the cforest function, this is not the case.) Therefore we suggest not to interpret the magnitude of the scaled variable importance of the randomForest function.

The plots show that for the randomForest function (cf. top row in Figures 3 and 4) and, less pronounced, for the cforest function with bootstrap sampling (cf. bottom row, left plot in Figures 3 and 4), the deviation of the permutation importance measure over the simulation runs is highest for the variable *X*_5 _with the highest number of categories, and decreases for the variables with less categories and the continuous variable. This effect is weakened but not substantially altered by scaling the measure (cf. Figure 3 vs. Figure 4).

As opposed to the obvious effect in the selection frequencies and the Gini importance, there is no effect in the mean values of the distributions of the permutation importance measures, which are in mean close to zero as expected for uninformative variables. However, the notable differences in the variance of the distributions for predictor variables with different scale of measurement or number of categories seriously affect the expressiveness of the variable importance measure.

In a single trial this effect may lead to a severe over- or underestimation of the variable importance of variables that have more categories as an artefact of the method, even though they are no more or less informative than the other variables.

Only when the cforest function is used together with subsampling without replacement (cf. bottom row, right plot in Figures 3 and 4) does the deviation of the permutation importance measure over the simulation runs not increase substantially with the number of categories or scale of measurement of the predictor variables.

Thus, only the variable importance measure available in cforest, and only when used together with sampling without replacement, reliably reflects the true importance of potential predictor variables in a scenario where the potential predictor variables vary in their scale of measurement or number of categories.

### Results of the power case simulation study

In the power case, where only the predictor variable *X*_2 _is informative, a sensible variable importance measure should be able to distinguish the informative predictor variable.

The following figures display the results of the power case with the highest value 0.2 of the *relevance *parameter, indicating a high degree of dependence between *X*_2 _and the response. In this setting, each of the variable importance measures should clearly prefer *X*_2_, while the respective values for the remaining predictor variables should be equally low.

**Figure 5:**
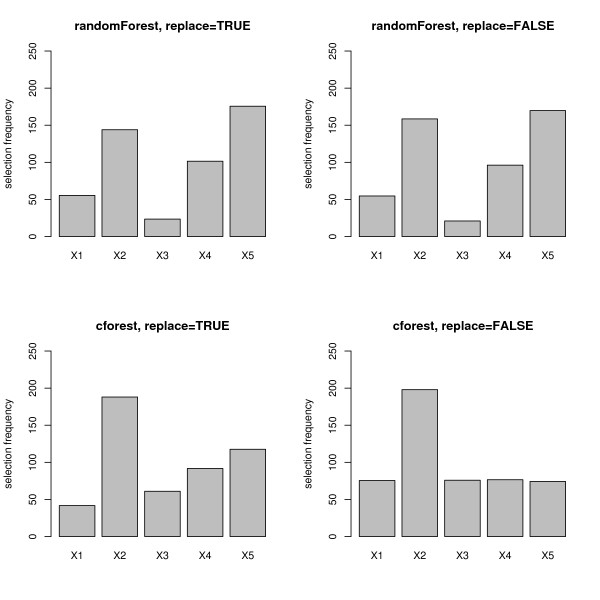
**Results of the power case study – variable selection frequency**. Mean variable selection frequencies for the power case, where only the second predictor variable is informative. The plots in the top row display the frequencies when the randomForest function is used, the bottom row when the cforest function is used. The left column corresponds to bootstrap sampling with replacement, the right column to subsampling without replacement.

Figure 5 shows that the mean selection frequencies (again over 1000 simulation runs) of the predictor variables again differ substantially when the randomForest function (cf. top row in Figure 5) is used, and the relevant predictor variable *X*_2 _cannot be identified. With the cforest function with bootstrap sampling (cf. bottom row, left plot in Figure 5) there is still bias obvious in the selection frequencies of the categorical predictor variables with many categories. Only when the cforest function is used together with subsampling without replacement (cf. bottom row, right plot in Figure 5), are the variable selection frequencies for the uninformative predictor variables equally low as desired, and the value for the relevant predictor variable *X*_2 _sticks out.

**Figure 6:**
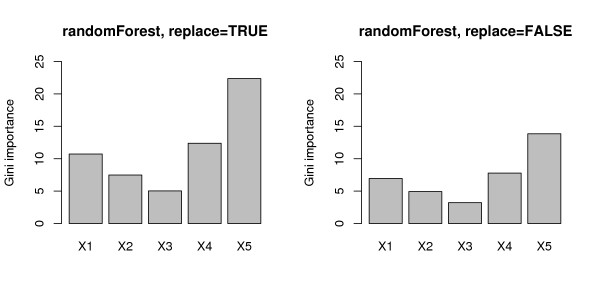
**Results of the power case study – Gini importance**. Mean Gini importance for the power case, where only the second predictor variable is informative. The left plot corresponds to bootstrap sampling with replacement, the right plot to subsampling without replacement.

The mean Gini importance, that is displayed in Figure 6, again shows a strong bias towards variables with many categories and the continuous variable. It completely fails to identify the relevant predictor variable, with the mean value for the relevant variable *X*_2 _only slightly higher than in the null case.

**Figure 7:**
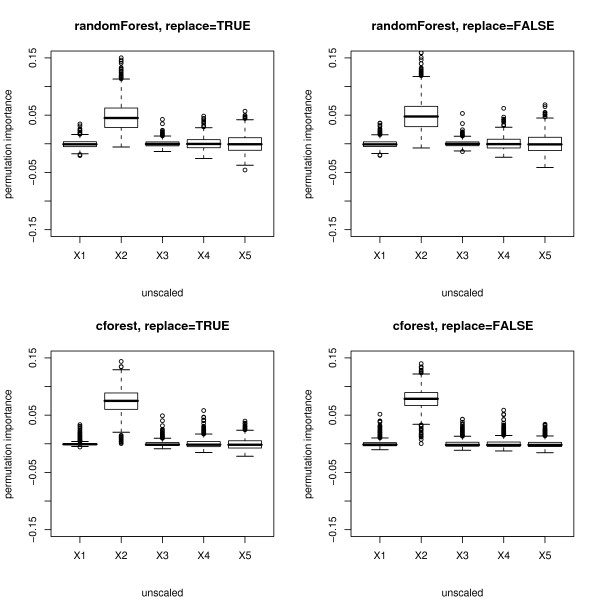
**Results of the power case study – unscaled permutation importance**. Distributions of the unscaled permutation importance measures for the power case, where only the second predictor variable is informative. The plots in the top row display the distributions when the randomForest function is used, the bottom row when the cforest function is used. The left column corresponds to bootstrap sampling with replacement, the right column to subsampling without replacement.

**Figure 8:**
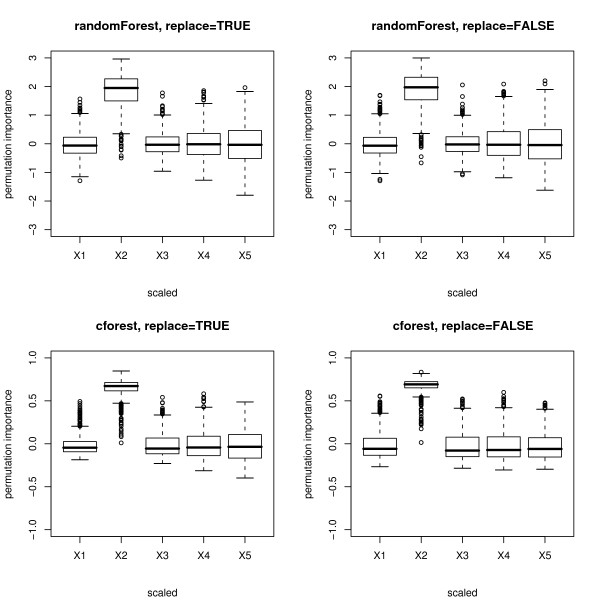
**Results of the power case study – scaled permutation importance**. Distributions of the scaled permutation importance measures for the power case, where only the second predictor variable is informative. The plots in the top row display the distributions when the randomForest function is used, the bottom row when the cforest function is used. The left column corresponds to bootstrap sampling with replacement, the right column to subsampling without replacement.

Figures 7 and 8 show boxplots of the distributions of the unscaled and scaled permutation importance measures of both functions. Again for the randomForest function (cf. top row in Figures 7 and 8) and, less pronounced, for the cforest function with bootstrap sampling (cf. bottom row, left plot in Figures 7 and 8), the deviation of the permutation importance measure over the simulation runs is highest for the variable *X*_5 _with the highest number of categories, and decreases for the variables with less categories and the continuous variable. This effect is weakened but not substantially altered by scaling the measure (cf. Figure 7 vs. Figure 8).

As expected the mean value of the permutation importance measure for the informative predictor variable *X*_2 _is higher than for the uninformative variables. However, the deviation of the variable importance measure for the uninformative variables with many categories *X*_4 _and *X*_5 _is so high that in a single trial these uninformative variables may outperform the informative variable as an artefact of the method. Thus, only the variable importance measure computed with the cforest function, and only when used together with sampling without replacement, is able to reliably detect the informative variable out of a set of uninformative competitors, even if the degree of dependence between *X*_2 _and the response is high. The rate at which the informative predictor variable is correctly identified (by producing the highest value of the permutation importance measure) increases with the degree of dependence between *X*_2 _and the response. In Table 3 the rates of correct identifications (over 1000 simulation runs) for four different degrees of dependence between *X*_2 _and the response are summarized for the randomForest and cforest function with different options.

**Table 3:** Rates of correct identifications of the informative variable for the power case

			Degree of dependence			
	Method	Replacement	0.05	0.1	0.15	0.2
Scaled	randomForest	true	0.234	0.497	0.770	0.956
		false	0.237	0.489	0.760	0.949
	cforest	true	0.338	0.672	0.923	0.991
		false	0.365	0.728	0.943	0.994

Unscaled	randomForest	true	0.194	0.413	0.701	0.928
		false	0.186	0.400	0.710	0.919
	cforest	true	0.324	0.648	0.910	0.989
		false	0.370	0.729	0.943	0.994

Rates of correct identifications of the informative variable with the scaled and unscaled permutation importance of the randomForest method, applied with sampling with and without replacement, as compared to those of the cforest method, applied with sampling with and without replacement, as a function of the degree of dependence (indicated by the *relevance *parameter, cf. Table 2) between the informative variable *X*_2 _and the response. (Standard errors of the rates of correct identifications *r *over 1000 iterations can easily be computet by *se *= r⋅(1−r)/1000
 MathType@MTEF@5@5@+=feaafiart1ev1aaatCvAUfKttLearuWrP9MDH5MBPbIqV92AaeXatLxBI9gBaebbnrfifHhDYfgasaacH8akY=wiFfYdH8Gipec8Eeeu0xXdbba9frFj0=OqFfea0dXdd9vqai=hGuQ8kuc9pgc9s8qqaq=dirpe0xb9q8qiLsFr0=vr0=vr0dc8meaabaqaciaacaGaaeqabaqabeGadaaakeaadaGcaaqaaiabdkhaYjabgwSixlabcIcaOiabigdaXiabgkHiTiabdkhaYjabcMcaPiabc+caViabigdaXiabicdaWiabicdaWiabicdaWaWcbeaaaaa@3A1A@.)

For all degrees of dependence between *X*_2 _and the response *Y *the cforest function detects the informative variable more reliably than the randomForest function, and the cforest function used with subsampling without replacement outperforms the cforest function with bootstrap sampling with replacement. For the randomForest function scaling the permutation importance measure can slightly increase the rates of correct identifications because, as shown in Figures 4 and 8, scaling weakens the differences in variance of the permutation importance measure for variables of different scale of measurement and number of categories. For the cforest function, that is not affected by the scale of measurement and number of categories of the predictor variables, both the unscaled and the scaled permutation importance perform equally well.

So far we have seen that for the assessment of variable importance and variable selection purposes it is important to use a reliable method, that is not affected by other characteristics of the predictor variables. Statistical explanations of our findings are given in a later section.

In addition to its superiority in the assessment of variable importance the cforest method, especially when used together with subsampling without replacement, can also be superior to the randomForest method with respect to classification accuracy in situations like that of the power case simulation study, where uninformative predictor variables with many categories "fool" the randomForest function.

Due to its artificial preference for uninformative predictor variables with many categories the randomForest function can produce a higher mean misclassification rate than the cforest function. The mean misclassification rates (again over 1000 simulation runs) for the randomForest and cforest function, again for four different degrees of dependence and used with sampling with and without replacement, are displayed in Table 4.

**Table 4:** Mean misclassification rates for the power case

		Degree of dependence			
Method	Replacement	0.05	0.1	0.15	0.2
randomForest	true	0.4945 (0.0014)	0.4819 (0.0015)	0.4510 (0.0016)	0.4028 (0.0017)
	false	0.4942 (0.0014)	0.4814 (0.0015)	0.4496 (0.0016)	0.4026 (0.0017)
cforest	true	0.4910 (0.0014)	0.4660 (0.0016)	0.4169 (0.0019)	0.3491 (0.0019)
	false	0.4879 (0.0014)	0.4581 (0.0017)	0.4022 (0.0019)	0.3384 (0.0019)

Mean misclassification rates of the randomForest method, applied with sampling with and without replacement, as compared to those of the cforest method, applied with sampling with and without replacement, as a function of the degree of dependence (indicated by the *relevance *parameter, cf. Table 2) between the informative variable *X*_2 _and the response. (Standard errors of the mean misclassification rates are given in parentheses.)

Each method was applied to the same simulated test set in each simulation run. The test sets were generated from the same data generating process as the learning sets. We find that for all degrees of dependence between *X*_2 _and the response *Y *the cforest function, especially with sampling without replacement, outperforms the other methods. A similar result is obtained in the application to C-to-U conversion data presented in the next section.

The differences in classification accuracy are moderate in the latter case, however one could think of more extreme situations that would produce even greater differences. This shows that the same mechanisms underlying the variable importance bias can also affect the classification accuracy, e.g. when suboptimal predictor variables, that do not add to the classification accuracy, are artificially preferred in variable selection merely because they have more categories.

### Application to C-to-U conversion data

RNA editing is the process whereby RNA is modified from the sequence of the corresponding DNA template [11]. For instance, cytidine-to-uridine conversion (abbreviated C-to-U conversion) is common in plant mitochondria. The mechanisms of this conversion remain largely unknown, although the role of neighboring nucleotides is emphasized. Cummings and Myers [11] suggest to use information from sequence regions flanking the sites of interest to predict editing in *Arabidopsis thaliana*, *Brassica napus *and *Oryza sativa *based on random forests. The *Arabidopsis thaliana *data of [11] can be loaded from the journal's homepage. For each of the 876 observations, the data set gives

• the response at the site of interest (binary: edited/not edited) and as potential predictor variables

• the 40 nucleotides at positions -20 to 20, relative to the edited site (4 categories),

• the codon position (4 categories),

• the estimated folding energy (continuous) and

• the difference in estimated folding energy between pre-edited and edited sequences (continuous).

**Figure 9:**
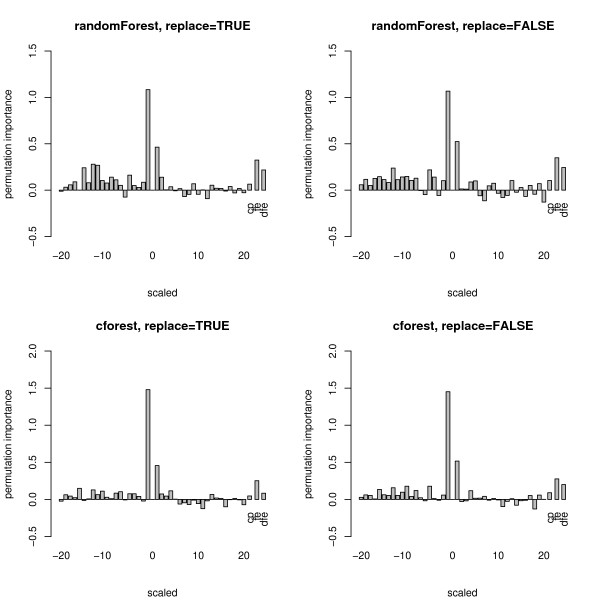
**Results for the C-to-U conversion data – scaled permutation importance**. Scaled variable importance measures for the C-to-U conversion data. The plots in the top row display the measures when the randomForest function is used, the bottom row when the cforest function is used. The left column corresponds to bootstrap sampling with replacement, the right column to subsampling without replacement. In each plot the positions -20 through 20 indicate the nucleotides flanking the site of interest, and the last three bars on the right refer to the codon position (cp), the estimated folding energy (fe) and the difference in estimated folding energy (dfe).

We first derive the permutation importance measure for each of the 43 potential predictor variables with each method. As can be seen from the barplot in Figure 9, the (scaled) variable importance measures largely reflect the results of [11] based on the Gini importance measure, but differ slightly for the randomForest and cforest function and the different resampling schemes. In particular, the variable importance measure of the randomForest function seems to produce more "noise" than that of the cforest function: the contrast of amplitudes between irrelevant and relevant predictors is more pronounced when the cforest function is used.

Note, however, that the the permutation importance values for one predictor variable can vary between two computations, because each computation is based on a different random permutation of the variable. Therefore, before interpreting random forest permutation importance values, the analysis should be repeated (with several different random seeds) to test the stability of the results.

Similarly to the simulation study, we also compared the prediction accuracy of the four approaches for this data set. To do so, we split the original data set into learning and test sets with size ratio 2:1 in a standard split-sample validation scheme. A random forest is grown based on the learning set and subsequently used to predict the observations in the test set. This procedure is repeated 100 times, and the mean misclassification rates over the 100 runs are reported in Table 5. Again we find a slight superiority of the cforest function, especially when sampling is conducted without replacement. (Differences to the accuracy values reported in [11] are most likely due to their use of a different validation scheme, that is not reported in detail in [11].)

**Table 5:** Mean misclassification rates for application to C-to-U conversion data

Method	Replacement	
randomForest	true	0.2896 (0.0022)
	false	0.2879 (0.0026)
cforest	true	0.2807 (0.0024)
	false	0.2788 (0.0025)

Mean misclassification rates of the randomForest method applied with sampling with and without replacement as compared to those of the cforest method applied with sampling with and without replacement. (Standard errors of the mean misclassification rates are given in parentheses.)

All function calls and all important options of the randomForest and cforest functions used in the simulation studies and the application to C-to-U conversion data are documented in the supplement [see Additional file 1].

### Sources of variable importance bias

The main difference between the randomForest function, based on CART trees [18], and cforest function, based on conditional inference trees [29], is that in randomForest the variable selection in the individual CART trees is biased, so that e.g. variables with more categories are preferred. This is illustrated in the next section on variable selection bias in individual classification trees.

However, even if the individual trees select variables in an unbiased way as in the cforest function, we find that the variable importance measures, as well as the selection frequencies of the variables, are affected by the bootstrap sampling with replacement. This is explained in the section on effects induced by bootstrapping.

### Variable selection bias in the individual classification trees of a random forest

Let us again consider the null case simulation study design, where none of the variables is informative, and thus should be selected with equally low probabilities in a classification tree.

In traditional classification tree algorithms, like CART, for each variable a split criterion like the "Gini index" is computed for all possible cutpoints within the range of that variable. The variable selected for the next split is the one that produced the highest criterion value overall, i.e. in its best cutpoint.

Obviously variables with more potential cutpoints are more likely to produce a good criterion value by chance, as in a multiple testing situation. Therefore, if we compare the highest criterion value of a variable with two categories, say, that provides only one cutpoint from which the criterion was computed, with a variable with four categories, that provides seven cutpoints from which the best criterion value is used, the latter is often preferred. Because the number of cutpoints grows exponentially with the number of categories of unordered categorical predictors we find a preference for variables with more categories in CART-like classification trees. For further reading on variable selection bias in classification trees see, e.g., the corresponding sections in [24,25,28,29,33-35].

Since the Gini importance measure in randomForest is directly derived from the Gini index split criterion used in the underlying individual classification trees, it carries forward the same bias, as was shown in Figures 2 and 6.

Conditional inference trees [29], that are used to construct the classification trees in cforest, are unbiased in variable selection. Here, the variable selection is conducted by minimizing the p value of a conditional inference independence test, comparable e.g. to the *χ*^2 ^test, that incorporates the number of categories of each variable in the degrees of freedom.

**Figure 10:**
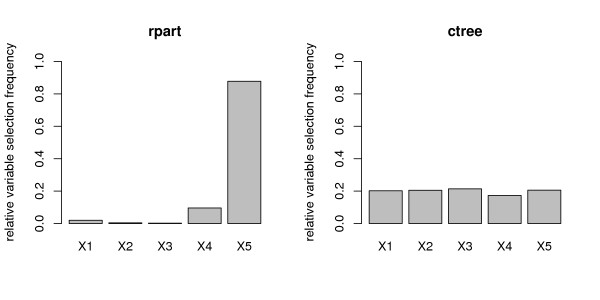
**Variable selection bias in individual trees**. Relative selection frequencies for the rpart (left) and the ctree (right) classification tree methods. All variables are uninformative as in the null case simulation study.

The mean selection frequencies (again over 1000 simulation runs) of the five predictor variables of the null case simulation study design for both CART classification trees (as implemented in the rpart function [36]) and conditional inference trees (function ctree) are displayed in Figure 10. We find that the variable selection with the rpart function is highly biased, while for the ctree function it is unbiased.

The variable selection bias that occurs in every individual tree in the randomForest function also has a direct effect on the variable importance measures of this function. Predictor variables with more categories are artificially preferred in variable selection in each splitting decision. Thus, they are selected in more individual classification trees and tend to be situated closer to the root node in each tree.

The variable selection bias affects the variable importance measures in two respects. Firstly, the variable selection frequencies over all trees are directly affected by the variable selection bias in each individual tree. Secondly, the effect on the permutation importance is less obvious but just as severe.

When permuting the variables to compute their permutation importance measure, the variables that appear in more trees and are situated closer to the root node can affect the prediction accuracy of a larger set of observations, while variables that appear in fewer trees and are situated closer to the bottom nodes affect only small subsets of observations. Thus, the range of possible changes in prediction accuracy in the random forest, i.e. the deviation of the variable importance measure, is higher for variables that are preferred by the individual trees due to variable selection bias.

We found in Figures 1 through 9, that the effects induced by the differences in scale of measurement of the predictor variables were more pronounced for the randomForest function, where variable selection in the individual trees is biased, than for the cforest function, where the individual trees are unbiased. However, we also found that when the cforest function is used with bootstrap sampling, the variable selection frequencies of the categorical predictors still depend on their number of categories (cf., e.g., bottom row, left plot in Figure 1), and also the deviation of the permutation importance measure is still affected by the number of categories (cf., e.g., bottom row, left plot in Figures 3 and 4).

Thus, there must be another source of bias, besides the variable selection bias in the individual trees, that affects the selection frequencies and the deviation of the permutation importance measure.

We show in the next section that this additional effect is due to bootstrap sampling with replacement, that is traditionally employed in random forests.

### Effects induced by bootstrapping

From the comparison of left and right columns (representing sampling with and without replacement) in Figures 1 and 5 we learned that the variable selection frequencies in random forest functions are affected by the resampling scheme.

We found that, even when the cforest function based on unbiased classification trees is used, variables with more categories are preferred when bootstrap sampling is conducted with replacement, while no bias occurs when subsampling is conducted without replacement, as displayed in the bottom right plot in Figures 1 and 5. Thus, the bootstrap sampling induces an effect that is more pronounced for predictor variables with more categories.

For a better understanding of the underlying mechanism let us consider only the categorical predictor variables *X*_2 _through *X*_5 _with different numbers of categories from the null case simulation study design.

Rather than trying to explain the effect of bootstrap sampling in the complex framework of random forests, we use a much simpler independence test for the explanation.

We consider the p values of *χ*^2 ^tests (computed from 1000 simulated data sets). In each simulation run, a *χ*^2 ^test is computed for each predictor variable and the binary response *Y*. Remember that the variables in the null case are not informative, i.e. the response is independent of all variables.

For independent variables the distribution of the p values of the *χ*^2 ^test is supposed to form a uniform distribution.

**Figure 11:**
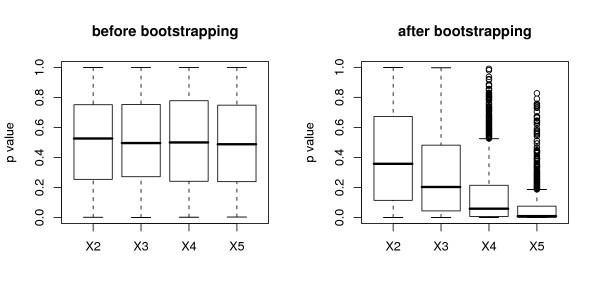
**Effects induced by bootstrapping**. Distribution of the p values of *χ*^2 ^tests of each categorical variable *X*_2_,..., *X*_5 _and the binary response for the null case simulation study, where none of the predictor variables is informative. The left plots correspond to the distribution of the p values computed from the original sample before bootstrapping. The right plots correspond to the distribution of the p values computed for each variable from the bootstrap sample drawn with replacement.

The left plot in Figure 11 displays the distribution of the p values of *χ*^2 ^tests from each predictor variable and the response *Y *as boxplots. We find that the boxplots range from 0 to 1 with median 0.5 as expected, because the p values of the *χ*^2 ^test form a uniform distribution when computed before bootstrapping. However, if in each simulation run we draw a bootstrap sample from the original sample and then again compute the p values based on the bootstrap sample, we find that the distribution of the p values is shifted towards zero as displayed in the right plot in Figure 11.

Obviously, the bootstrap sampling artificially induces an association between the variables. This effect is always present when statistical inference, such as an association test, is carried out on bootstrap samples: Bickel and Ren [37] point out that bootstrap hypothesis testing fails whenever the distribution of any statistic in the bootstrap sample, rather than the distribution of the statistic under the null hypothesis, is used for statistical inference. We found that this issue directly affects variable selection in random forests, because the deviation from the null hypothesis is more pronounced for variables that have more categories. The reason for the shift in the distribution of the p values displayed in Figure 11 is that each original sample, even if sampled from theoretically independent distributions, may show some minor variations from the null hypothesis of independence. These minor variations are aggravated by bootstrap sampling with replacement, because the cell counts in the contingency table are affected by observations that are either not included or are doubled or tripled in the bootstrap sample, and therefore the bootsrap sample deviates notably from the null hypothesis – even if the original sample was generated under the null hypothesis.

This effect is more pronounced for variables with more categories, because in larger tables (such as the 4 × 2 table from the cross-classification of *X*_3 _and the binary response *Y*), the absolute cell counts are smaller than in smaller tables (such as the 2 × 2 table from the cross-classification of *X*_2 _and the binary response *Y*). With respect to the smaller absolute cell counts, excluding or duplicating an observation produces more severe variations from the null hypothesis.

This effect is not eliminated if the sample size is increased, because in bootstrap sampling the size *n *of the original sample and the bootstrap sample size *n *increase simultaneously. However, if subsamples are drawn without replacement the effect disappears.

The apparent association that is induced by bootstrap sampling, and that is more pronounced for predictor variables with many categories, affects both variable importance measures: The selection frequency is again directly affected, and the permutation importance is affected because variables with many categories are selected more often and gain positions closer to the root node in the individual trees. Together with the mechanisms described in the previous section, this explains our findings.

From our simulation results we can see, however, that the effect of bootstrap sampling is mostly superposed by the much stronger effect of variable selection bias when comparing the conditions of sampling with and without replacement for the randomForest function only (cf. Figures 1 through 9, top row). Only when variable selection bias is removed by the cforest function the differences between the conditions of sampling with and without replacement are obvious (cf. Figures 1 through 9, bottom row). We therefore conclude that in order to be able to reliably interpret the variable importance measures of a random forest, the forest must be built from unbiased classification trees, and sampling must be conducted without replacement.

## Conclusion

Random forests are a powerful statistical tool, that has found many applicants in various scientific areas. It has been applied to such a wide variety of problems as large-scale association studies for complex genetic diseases, the prediction of phenotypes based on amino acid or DNA sequences, QSAR modeling and clinical medicine, to name just a few.

Features that have added to the popularity of random forests especially in bioinformatics and related fields, where identifying a subset of relevant predictor variables from very large sets of candidates is the major challenge, include its ability to deal with critical "small *n *large *p*" data sets and the variable importance measures it provides for variable selection purposes.

However, when a method is used for variable selection, rather than prediction only, it is particularly important that the value and interpretation of the variable importance measure actually depict the importance of the variable, and are not affected by any other characteristics.

We found that for the original random forest method the variable importance measures are affected by the number of categories and scale of measurement of the predictor variables, which are no direct indicators of the true importance of the variable.

As long as, e.g., only continuous predictor variables, as in most gene expression studies, or only variables with the same number of categories are considered in the sample, variable selection with random forest variable importance measures is not affected by our findings. However, in studies where continuous variables, such as the folding energy, are used in combination with categorical information from the neighboring nucleotides, or when categorical predictors, as in amino acid sequence data, vary in their number of categories present in the sample variable selection with random forest variable importance measures is unreliable and may even be misleading.

Especially informations on clinical and environmental variables are often gathered by means of questionnaires, where the number of categories can vary between questions. The number of categories is typically determined by many different factors, but is not necessarily an indicator of variable importance. Similarly, the number of different categories of a predictor actually available in a certain sample is not an indicator of its relevance for predicting the response. Hence, the number of categories of a variable should not influence its estimated importance – otherwise the results of a study could easily be distorted when an irrelevant variable with many categories is included in the study design.

We showed that, due to variable selection bias in the individual classification trees and effects induced by bootstrap sampling, the variable importance measures of the randomForest function are not reliable in many scenarios relevant in applied research.

As an alternative random forest method we propose to use the cforest function, that provides unbiased variable selection in the individual classification trees. When this method is applied with subsampling without replacement the resulting variable importance measure can be used reliably for variable selection even in situations where the potential predictor variables vary in their scale of measurement or their number of categories.

With respect to computation time the cforest function is more expensive than the randomForest function, because in order to be unbiased split decisions and stopping rely on time-consuming conditional inference. To give an impression, the computation times of the application to C-to-U conversion data, with 876 observations and 44 predictor variables, as stated in the supplementary file for the cforest function used with bootstrap sampling with replacement are in the range of 8.38 sec., while subsampling without replacement is computationally less expensive and in the range of 4.82.

Since we saw that only subsampling without replacement guarantees reliable variable selection and produces unbiased variable importance measures, the faster version without replacement should be preferred anyway. The computation time for the randomForest function is in the range of 0.24 sec. with and 0.18 sec. without replacement. However, we saw that the randomForest function should not be used when the potential predictor variables vary in their scale of measurement or their number of categories. The aim of this paper was to explore the limits of the empirical measures of variable importance provided for random forests, to understand the underlying mechanisms and to use that understanding to guarantee unbiased and reliable variable selection in random forests.

In a more theoretical work van der Laan [38] gives a fundamental definition of variable importance, as well as a statistical inference framework for estimating and testing variable importance. Inspired by this approach, future research on variable importance measures for variable selection with random forests aims at providing further means of statistical inference, that can be used to guide the decision on which and how many predictor variables to select in a certain problem.

## Authors' contributions

CS first observed the variable selection bias in random forests, set up and performed the simulation experiments, studied the sources of the selection bias and drafted the manuscript. ALB, AZ and TH contributed to the design of the simulation experiments, to theoretical investigations of the problem, and to the manuscript. TH implemented the cforest, ctree and varimp functions. ALB analyzed the C-to-U conversion data. All authors read and approved the final manuscript.

## Supplementary Material

Additional File 1 **R source code**. The exemplary R source code includes all function calls and comments on all important options of the randomForest and cforest functions, that were used in the simulation studies and the application to C-to-U conversion data. Please install the latest versions of the packages randomForest and party before use. Click here for file
